# Monitoring of gene knockouts: genome-wide profiling of conditionally essential genes

**DOI:** 10.1186/gb-2007-8-5-r87

**Published:** 2007-05-22

**Authors:** Lisa K Smith, Maria J Gomez, Konstantin Y Shatalin, Hyunwoo Lee, Alexander A Neyfakh

**Affiliations:** 1 Center for Pharmaceutical Biotechnology, University of Illinois, Chicago, Illinois 60607, USA; 2 Current address: Department of Biochemistry, New York University School of Medicine, New York, New York 10016, USA; 3 Deceased (20 April 2006)

## Abstract

We have developed a new microarray-based genetic technique, named MGK (Monitoring of Gene Knockouts), for genome-wide identification of conditionally essential genes. MGK identified bacterial genes that are critical for fitness in the absence of aromatic amino acids, and was further applied to identify genes whose inactivation causes bacterial cell death upon exposure to the bacteriostatic antibiotic chloramphenicol. Our findings suggest that MGK can serve as a robust tool in functional genomics studies.

## Abstract 2

Monitoring of gene knockouts is a new microarray-based genetic technique used for genome-wide identification of conditionally essential genes in bacteria

## Background

A major aim of modern biology is to establish a functional framework that relates genes and their products to biologic effects. Although much progress has been made in addressing this challenge, large gaps remain in our understanding of the function and 'purpose' of many genes in even the most well studied model organisms. For instance, only 54% of *Escherichia coli *genes have currently been functionally characterized based on experimental evidence [1]. The fraction of genes that have well understood functions is even smaller for less 'popular' experimental models.

Assessing the contribution of a particular gene product to the welfare of the cell is an intrinsically difficult task to perform on a genome-wide scale. The process can be greatly expedited by employing two key experimental resources: first, comprehensive collections of knockout mutants; and second, a rapid and accurate means to determine the fitness of all mutants in parallel under given experimental conditions. Since the introduction of global transposon mutagenesis and gene replacement techniques, gene knockout mutant collections for a variety of micro-organisms have been generated, and many more are in progress. However, robust methods to monitor the fitness of mutants in mixed populations have been elusive; although selecting for enrichment of mutants is relatively easy, it is much more difficult to identify 'unfit' mutants that become depleted after selection.

To address this problem, we developed a simple and robust method, named MGK (Monitoring of Gene Knockouts), for the rapid identification of genes that contribute to bacterial fitness in various selective conditions. MGK uses flanking sequences of inserted antibiotic cassette (used for inactivation of a gene) as identifiers of mutants and allows simultaneous monitoring of thousands of mutants in a mixed library. In a model MGK screen, we successfully identified all 13 known genes whose inactivation confers aromatic amino acid auxotrophy on *E. coli*. The utility of MGK was further verified by identifying genes whose disruption resulted in bacterial cell death in the presence of the bacteriostatic antibiotic chloramphenicol. The versatility of MGK was demonstrated by applying it to two principally different gene knockout libraries: random transposon insertion library and genetic replacement library.

## Results

### Principle of MGK

**Figure 1:**
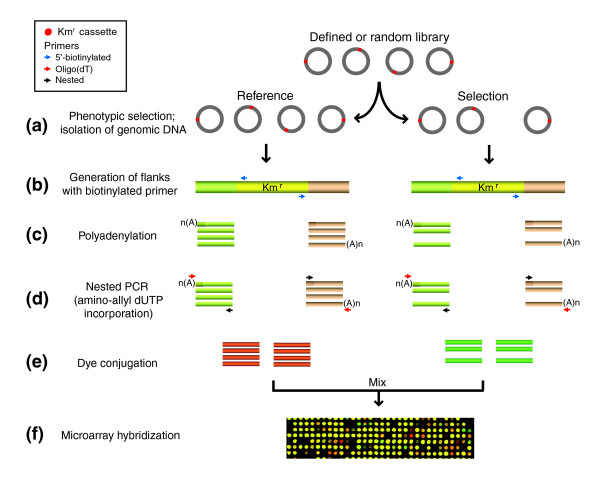
Schematic representation of MGK. **(a) **Mixed library is grown in a reference and selective condition, and genomic DNA is isolated from each population. **(b) **Using genomic DNA as template, single-stranded DNA flanks are generated by linear extension of outward-facing insertion cassette-specific biotinylated primers (blue arrows). **(c) **The biotinylated flanks are separated from the template using streptavidin-coated magnetic beads, and polyadenylated at the 3'-ends using terminal deoxynucleotidyl transferase in the presence of dATP. **(d) **Microarray targets are PCR-amplified using an oligo d(T) primer (red arrows) and a nested Km^r^-specific primer (black arrows). Amino-allyl dUTP is incorporated during this step. **(e) **Fluorescent dyes are conjugated to microarray targets. **(f) **Differentially labeled targets are mixed and hybridized to a custom DNA microarray. Km^r^, kanamycin resistance; MGK, Monitoring of Gene Knockouts; PCR, polymerase chain reaction.

MGK simultaneously tracks the relative abundance of individual mutants in gene inactivation libraries grown in a reference and experimental condition. This is achieved by hybridizing polymerase chain reaction (PCR)-amplified flanks of the inactivated genes to specifically designed DNA microarrays (Figure 1). The approach utilizes random or defined gene knockout libraries, in which individual genes are inactivated by either transposon insertion or gene replacement (kanamycin resistance [Km^r^] cassette in Figure 1).

The mixed library of knockout mutants is grown in a reference and selective condition for several generations. Genomic DNA isolated from each population serves as a template in a primer extension reaction. The chromosomal regions flanking the gene replacement cassette or sites of the transposon insertion are linearly amplified by repeated rounds of time-controlled extension of biotinylated primers specific for the Km^r ^cassette (Table 1). The yield of amplified flanks of the Km^r ^cassette corresponds to the relative abundance of individual gene knockout mutants in the population. Streptavidin-coated magnetic beads are used to isolate the biotinylated flanks, which are polyadenylated at the 3'-ends using terminal deoxynucleotidyl transferase. The flanks are then exponentially PCR amplified using a nested Km^r ^cassette-specific primer and an oligo-dT primer, yielding 'MGK targets'. At this step, amino-allyl modified dUTP is incorporated into the MGK targets and subsequently conjugated with fluorescent dyes. A mixture of labeled targets is then hybridized to a custom designed oligonucleotide microarray, and the relative abundance of individual mutants present in the library after growth in the reference and selective conditions is assessed. Once a preliminary list of conditionally essential genes is generated, the phenotypes of individual mutants (either picked from the arrayed gene inactivation defined libraries or engineered *de novo *in the case of random transposon insertion libraries) is verified.

**Table 1:** Primers used in this study

Primer name	Sequence
For defined library	

Up-BIO	5'-biotin-GAACTTCGAACTGCAGGTCGAC-3'
Dn-BIO	5'-biotin-GTATAGGAACTTCGAAGCAGCTC-3'
Up-Cy3	5'-GGTC**G**ACGGA**C**CCCCG-3'
Up-Cy5	5'-GGTC**A**ACGGA**T**CCCCG-3'
Dn-Cy3	5'-AAGCAG**T**TCCAG**C**CTACA-3'
Dn-Cy5	5'-AAGCAG**C**TCCAG**T**CTACA-3'

For random library	

Tn10OE-BIO	5'-biotin-CAAGATGTGTATCCACCTTAACTTAA-3'
Tn10OE OUT-Cy3	5'-ACCAA**T**ATCAT**T**AGGGGAT-3'
Tn10OE OUT-Cy5	5'-ACCAA**A**ATCAT**A**AGGGGAT-3'

Common for both libraries	

TATV-3 or Oligo(dT_9 _AT_15_V)	5'-T_9 _AT_15_V-3'
TATV-5 or Oligo(dT_15 _AT_9_V)	5'-T_15 _AT_9_V-3'

Mismatched nucleotides are in bold. 'V' represents A, C, or G.

The design of the DNA microarray for MGK depends on the type of mutant library being analyzed. For the *E. coli *random transposon insertion library employed in this study, a microarray was designed to contain unique oligonucleotide sequences (34-mer, on average) spaced approximately every 500 base pairs (bp) in the *E. coli *genome. As a result, each gene knockout was represented by one to three probes. For the *E. coli *defined deletion mutant library, 34-mer oligonucleotide sequences were selected from a region about 100 bp upstream and 100 bp downstream of each gene, so that each knockout was represented by two flanking probes. For clarity, the random transposon library and defined deletion library used in this study are referred to as 'random library' and 'defined library', respectively.

### MGK readily identifies genes of a known biochemical pathway

**Figure 2:**
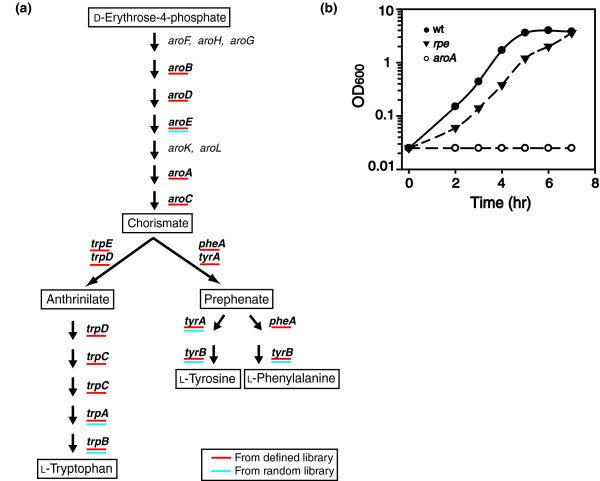
Genes identified by MGK as essential for cell growth in the absence of aromatic amino acids. **(a) **Biosynthetic pathway of aromatic amino acids in *E. coli*. Shown in bold are the 13 genes whose inactivation is expected to cause aromatic amino acid auxotrophy. Genes *aroF*, *aroH*, *aroG*, *aroK*, and *aroL *are involved in parallel biochemical routes and their disruption should not cause auxotrophy. Underlined in red are genes identified by MGK with the defined library, and in blue with the random library. **(b) **Growth of select mutants in defined medium lacking aromatic amino acids. The behavior of *aroB*, *aroC*, *aroD*, *aroE*, *epd*, *pheA*, *pdxA*, *tktA*, *trpA*, *trpB*, *trpC*, *trpD*, *trpE*, *tyrA*, and *tyrB *mutants identified by MGK screen were essentially indistinguishable from *aroA*. Growth of *ygdD *mutant was similar to *rpe *mutant. Supplementing the medium with aromatic amino acids restored growth of all mutants to wild-type level. Supplementing the medium with vitamin B_6 _restores growth of *epd *and *pdxA *mutants (data not shown). MGK, Monitoring of Gene Knockouts; OD, optical density; wt, wild-type *Escherichia coli*.

The aromatic amino acid biosynthesis pathway has been well characterized in *E. coli *[2]. Decades of painstaking experiments have identified 18 genes that are involved in the production of aromatic amino acids when they are not readily available in the environment (Figure 2a). Thirteen genes belonging to this pathway encode nonredundant enzymes and are expected to be essential for cell growth in medium lacking phenylalanine, tryptophan, and tyrosine. To evaluate the applicability of MGK for identification of conditionally essential genes, we used MGK to identify mutants (in both a random and a defined library) that are unable to grow in medium lacking aromatic amino acids. The *E. coli *random library of about 1.2 × 10^5 ^mutants was generated using random mini-Tn10 transposon mutagenesis [3]. The defined library consisted of 3,985 *E. coli *gene replacement mutants [4]; mutants in this library were mixed at equal ratio (see Materials and methods, below). In addition to demonstrating the flexibility of the method, the use of two types of libraries provided an opportunity to test the versatility of MGK and assess the extent to which mutant representation affected the sensitivity of the MGK screen.

For the MGK selection, libraries were grown for 10 generations in defined medium either containing or lacking aromatic amino acids (see Materials and methods, below, for details). MGK targets were prepared from each library and hybridized to corresponding microarrays. Experiments were performed twice, with dye swapping (correlation coefficient between two experiments was 0.84 for the defined library and 0.93 for the random library). (For the entire set of microarray raw data and intensity ratios, see Additional data files 1 and 2.)

Using the cut-off criteria described in Materials and methods (below), eight genes were identified as putatively essential for *E. coli *growth in the absence of aromatic amino acids in the random library, and 37 genes were identified from the defined library (Table 2). As mentioned above, there are 13 genes whose inactivation is expected to cause aromatic amino acid auxotrophy in *E. coli *[2]. All 13 of these genes were among the 37 genes identified in the MGK screen applied to the defined library, whereas five of the anticipated 13 auxotrophic mutants were among the eight genes found in the random library (Figure 2a). This finding demonstrates that MGK can successfully be applied to both types of libraries but that it provides a more complete dataset when it is used with the defined library.

**Table 2:** List of genes identified by MGK as important for growth in the absence of aromatic amino acids

Mutant library used	Genes				
Defined	*trpA*^a ^(28.1)	*pheA*^a ^(26.1)	*epd*^a ^(23.8)	*trpE*^a ^(22.8)	*aroE*^a ^(20.1)
	*tyrA*^a ^(17.5)	*aroC*^a ^(14.8)	*trpB*^a ^(13.9)	*aroA*^a ^(13.3)	*aroB*^a ^(11.9)
	*tyrB*^a ^(12.2)	*trpD*^a ^(7.4)	*ygcL *(6.6)	*rcsF *(6.3)	*yddK *(5.7)
	*ygaF *(5.4)	*ynaJ *(5.3)	*pdxA*^a ^(5.2)	*wzc *(5.0)	*surA *(4.9)
	*rpe*^a ^(4.8)	*ygdD*^a ^(4.7)	*aroF *(4.1)	*yadN *(4.0)	*uvrY *(4.0)
	*yeeA *(4.0)	*ydhH *(3.9)	*yfhD *(3.9)	*yadK *(3.8)	*ompX *(3.6)
	*aroD*^a ^(3.5)	*yfhM *(3.5)	*yedV *(3.4)	*ybeL *(3.4)	*trpC*^a ^(3.3)
	*wcaA *(3.3)	*ydhX *(3.2)			

Random	*tyrB*^a ^(15.9)	*epd*^a ^(13.3)	*trpA*^a ^(10.8)	*aroE*^a ^(10.4)	*tyrA*^a ^(7.8)
	*trpB*^a ^(3.9)	*hepA *(3.5)	*tktA*^a ^(3.2)		

Values in parentheses are intensity ratios (normalized reference intensity value/normalized selection intensity value), and are the average of two independent, inversely labeled experiments. All mutants were individually tested. ^a^Mutants that exhibited a growth defect in the absence of aromatic amino acids. MGK, Monitoring of Gene Knockouts.

Because several of the genes identified by MGK (three from the random library and 24 from the defined library) were previously unknown to be important for aromatic amino acid biosynthesis, the phenotypes of these gene deletion strains were tested. The disruption of *epd *and *pdxA*, found in the defined library, did cause a growth defect in medium lacking aromatic amino acids (Figure 2b). The encoded enzymes are involved in biosynthesis of pyridoxine (vitamin B_6_), which is an essential co-factor of transaminase steps in the aromatic biosynthesis pathway [5]. Finding these genes in our MGK screen was not surprising because the defined medium used in this study lacks vitamin B_6_. Indeed, growth of the *epd *and *pdxA *mutants was restored to wild-type levels in the medium supplemented with vitamin B_6 _(data not shown). Three other mutants, namely *tktA *identified in the random library, and *rpe *and *ygdD *found in the defined library, also exhibited reduced growth in medium lacking aromatic amino acids (Figure 2b). The encoded enzymes TktA (transketolase) [6] and Rpe (ribulose phosphate 3-epimerase) [7] are both involved in sugar phosphate interconversion in the nonoxidative branch of the pentose phosphate pathway; the function of YgdD is unknown. Although it is not clear why disruption of these genes reduces cell growth in the absence of aromatic amino acids, the phenotypes of these mutants confirm that they were legitimately identified by MGK. Disruption of the rest of the genes recovered in MGK screens (one from the random library and 20 from the defined library) caused no growth defect in the absence of aromatic amino acids, suggesting that either they were false-positive hits or that our conditions for testing individual mutants did not adequately reproduce the selection pressure experienced by mutants in the mixed library culture.

The results of this model MGK screen demonstrate that the method is well suited to genome-wide identification of conditionally essential genes. In addition, the comparison of results obtained from two libraries shows that the use of a defined library in which mutants are well represented increases the sensitivity of MGK screen.

### Identification of genes whose disruption leads to cell death upon exposure to a bacteriostatic antibiotic

Bacteriostatic antibiotics inhibit cell growth but they do not significantly decrease the number of viable cells. Proteins whose genetic knockout leads to bacterial cell death upon treatment with bacteriostatic antibiotics may serve as new targets for drug potentiators and may provide important insights into mechanisms of bacterial response to antibiotic stress. Identification of such mutations generally requires the near impossible task of plating thousands of mutant cultures onto multiple plates after exposing them to bacteriostatic antibiotics.

MGK provides a much better way to identify such mutants. As a proof of concept, we used MGK to identify genes required for survival of *E. coli *during challenge with chloramphenicol, which is a classic bacteriostatic antibiotic that prevents bacterial growth by interfering with the activity of the ribosomal peptidyl transferase [8]. The pooled defined library was exposed for two consecutive rounds of 18-hour incubations in the presence of chloramphenicol (80 μg/ml, which is ten times the minimum inhibitory concentration). MGK targets prepared from libraries with or without chloramphenicol selection were hybridized to the microarray. (For entire set of microarray raw data and intensity ratios, see Additional data file 3.)

**Figure 3:**
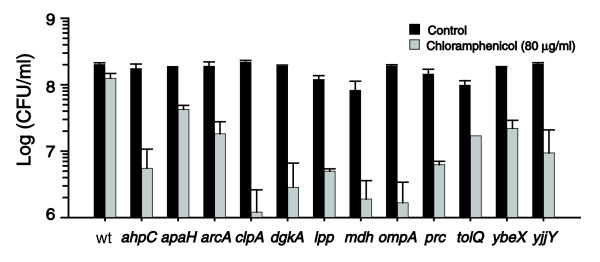
Decreased survival of mutants upon treatment with a bacteriostatic antibiotic chloramphenicol. Shown is the number of viable cells (colony forming units [CFU]) in 1 ml cell culture before addition of antibiotic (black bars) or after 18 hours of incubation in the presence of 80 μg/ml chloramphenicol (gray bars). Values shown are the average of two independent experiments. Error bars correspond to the standard deviation and are shown only if they are larger than the resolution of the figure. wt: wild-type *E. coli*.

We identified 35 genes that exhibited at least threefold reduced signal intensity after cells were exposed to chloramphenicol (Table 3). Some of the identified genes were known to be co-transcribed within an operon (*pstC *and *pstS*; *ptsH *and *ptsI*; *sufB *and *sufD*; and *tolQ*, *tolR*, and *tolA*), or their gene products constituted a functional pair (*arcA *and *arcB*). We verified the phenotypes by testing survival of 29 individual mutants upon chloramphenicol treatment (among these 29 mutants, each of the aforementioned operons were represented by one mutant). Of these, 12 mutants exhibited more than fivefold reduced number of viable cells after exposure to chloramphenicol, and therefore they carried deletions of genes that are critical for survival of bacteria upon treatment with a bacteriostatic antibiotic. In comparison, the viable cell count of the wild-type was not affected by chloramphenicol (Figure 3).

**Table 3:** List of genes identified by MGK as important for survival upon chloramphenicol treatment

Phenotype verification of mutants	Genes
Not tested	*pstS *(10.5), *tolA/B *(7.2), *ptsI *(4.9), *acrB *(4.2), *tolA/R *(4.2), *sufB *(3.4)
Individually tested mutants	*hns *(12.7), *dgkA *(11.5), *rnhA *(10.2), *apaH *(10.0), *rluD *(8.6), *ahpC *(8.3), *ompA *(8.1), *pstC *(7.7), *rfaE *(7.7), *arcA *(6.8), *yjjY *(6.7), *oxyR *(6.2), *gor *(5.5), *rfaF *(5.0), *sufD *(5.0), *lpp *(4.9), *prc *(4.7), *ybeX *(4.6), *fpr *(4.6), *acrA *(4.5), *tolQ *(4.5), *arcB *(4.0), *phoP *(3.9), *clpA *(3.7), *ydhD *(3.6), *mdh *(3.5), *yqiC *(3.5), *miaA *(3.4), *ptsH *(3.4)
Individually tested mutants exhibiting a fivefold or greater killing with 18-hour exposure to chloramphenicol	*dgkA *(11.5), *apaH *(10.0), *ahpC *(8.3), *ompA *(8.1), *arcA *(6.8), *yjjY *(6.7), *lpp *(4.9), *prc *(4.7), *ybeX *(4.6), *tolQ *(4.5), *arcB *(4.0), *clpA *(3.7), *mdh *(3.5)

Values in parentheses are intensity ratios (normalized reference intensity value/normalized selection intensity value), and are the average of two independent, inversely labeled experiments. In the case of *tolA*/*B *and *tolA*/*R*, the origin of signal intensity could not be distinguished between two neighboring genes. MGK, Monitoring of Gene Knockouts.

The functional categories of the verified 12 genes varied widely, including peroxide detoxification (AhpC), redox regulation (ArcA), proteolysis (ClpP and Prc), membrane integrity (Lpp), and transport (TolQ, OmpA, and YbeX). From this diverse set, we can only tentatively rationalize the importance of a few genes for cell survival upon antibiotic treatment (see Discussion, below). This finding underscores the advantage of an unbiased global gene-screening technique such as MGK for identifying potential new drug targets as well as targets for drug potentiators. Taken together, the results presented here demonstrate the power of MGK for identifying loss-of-function mutations in complex mutant libraries.

## Discussion

In this paper we present a new microarray-based technique, MGK, for monitoring genetic knockouts, as a general genomics approach to rapid identification of conditionally essential genes. The principle of MGK, namely using amplified flanks of the inactivated genes as identifiers of mutants, is shared with previously described techniques [9-11]. However, MGK has the valuable advantages of high robustness and a streamlined procedure that eliminates the need for *in vitro *transcription, ligation, and multiple PCRs [9-11]. Importantly, the method does not rely upon the presence of any specific element in the gene-inactivation cassette such as a T7 promoter [9,10] or molecular bar probes [12]; it requires only the synthesis of an insert-specific biotinylated DNA primer. Therefore, it can be applied to any existing gene knockout library (including such species as *Bacillus anthracis *[13], *Bacillus subtilis *[14], *Mycobacterium paratuberculosis *[15], *Neisseria meningitidis *[16], *Pseudomonas aeruginosa *[17,18], *Staphylococcus aureus *[19], and *Saccharomyces cerevisiae *[20], for which defined libraries are already available).

As proof of the concept, we demonstrated the ability of MGK to identify accurately the *E. coli *genes that are required for growth in the absence of aromatic amino acids. Employing the defined library, all of the 13 genes whose disruption is expected to cause auxotrophy were identified. Only five of these genes were identified when a random transposon knock-out library was used. The incomplete gene identification using the random library probably arose from a biased transposon distribution along the *E. coli *chromosome. We have evidence that, in our library, the frequency of transposon insertion was skewed in favor of chromosomal regions close to the origin of replication (Additional data files 4 and 5). Thus, although MGK can be applied to both random and defined gene-inactivation libraries, the selection carried out with the defined library provides a more comprehensive list of mutants with the desired phenotype.

Several additional factors make a defined gene-inactivation library a more favorable starting material for the MGK selection. In a defined library each gene is targeted individually for mutagenesis, which allows better representation of knockout mutants within a collection comprised of a limited number of strains (3,985 mutants in the Keio collection). With a random knockout library, even of a high complexity (1.2 × 10^5 ^in our random gene knockout library), the inactivation of every nonessential gene is never certain. In addition to uncertainty of saturation, that transposon insertion in a gene does not always result in functional inactivation complicates the analysis of random transposon mutants in a pool. Furthermore, the opportunity to use a collection with a smaller number of mutants without sacrificing comprehensiveness is advantageous for *in vivo *selections in which the size of the inoculum is limited. Another important benefit of utilizing defined collections of mutants for MGK studies is the ease of testing phenotypes of individual strains. Unlike random libraries, in which mutant strains are generated as a mixture, necessitating the re-engineering of each strain of interest, defined collections consist of strains that have already been individually archived.

We further verified the power of the MGK technique by identifying *E. coli *genes that are critical for bacterial survival during exposure to a bacteriostatic antibiotic chloramphenicol. Applying MGK, we identified 12 genes (*ahpC*, *apaH*, *arcA*, *clpA*, *dgkA*, *lpp*, *mdh*, *ompA*, *prc*, *tolQ*, *ybeX*, and *yjjY*), whose disruption was shown to cause cell death in the presence of chloramphenicol. The functions of several genes from this set are related to biosynthesis or structure of the bacterial envelope. These include *dgkA*, which encodes diacylglycerol kinase (involved in phospholipid turnover) [21]; *ompA*, which encodes an outer membrane porin [22]; *lpp*, which encodes an outer membrane protein anchoring the outer membrane to the peptidoglycan [2]; and *prc*, which encodes a periplasmic protease [23]. It is possible that the inhibition of protein synthesis by chloramphenicol weakens the cell envelope because of difference in stability between biosynthetic and metabolizing enzymes, and that this process is exacerbated in these mutants, which leads to cell death upon treatment with a protein synthesis inhibitor. We also found that disruption of *arcA *and *arcB*, which comprise the ArcAB two-component signal transduction system that is involved in regulation of aerobic respiration [24], as well as disruption of the gene *ahpC*, which encodes a subunit of alkylhydroperoxide reductase [25], led to cell death upon treatment with chloramphenicol. This finding may indicate that the ability to cope efficiently with oxidative stress is critical to bacterial survival upon cessation of translation. Analysis of these and other genes identified in the MGK screen is currently in progress. Products encoded in some of the identified genes may provide new insights into the mechanism of antibiotic action and interesting venues for developing antibiotic potentiators.

The results presented in this study clearly support the utility of MGK for simultaneous analysis of the relative fitness of a large number of mutants in a mixed culture, and therefore for identifying conditionally essential genes. However, like other genome-wide approaches, MGK is expected to yield a certain fraction of false positive hits. The direct testing of phenotypes of individual mutants appears to indicate that approximately half of the mutants we identified using the cut-off criteria described in the Materials and methods section (see below) were false positive. It should be noted, however, that when tested in monoculture, a mutant may exhibit growth characteristics different from those when it is grown in competition with other mutants. In general, the number of false-positive mutants can be further reduced by increasing the number of independent experiments, or using a more stringent cut-off value for the hybridization signal intensity ratio. However, if the list of identified genes is relatively small, then it is easy to test individual strains to confirm or refute predicted phenotypes when access to individual mutants is readily available (as in the defined libraries).

## Conclusion

In this paper we have described a new technique, MGK, which employs DNA microarrays to assess simultaneously the relative fitness of gene-inactivation mutants grown under selective conditions. As proof of principle, we have demonstrated the ability of MGK by identifying all 13 *E. coli *genes that are known to be required for growth in medium lacking aromatic amino acids. In addition, we applied MGK to identify genes that are critical for survival during treatment with a bacteriostatic antibiotic, namely chloramphenicol. Furthermore, we showed that although MGK can be applied to analysis of different types of inactivation libraries, the sensitivity of the screen improves with the comprehensiveness of mutant representation (as shown by comparison of the results of the screens performed with the defined and random libraries). The results presented in this study clearly demonstrate that MGK provides a rapid and accurate means to identify conditionally essential genes by simultaneously assessing the relative fitness of gene inactivation mutants in a complex collection.

The spectrum of possible applications of MGK is very broad. As a functional genomics tool, the method can facilitate characterization of genes with unknown functions, and may reveal new tasks of previously characterized genes. MGK can be applied to the identification of drug targets and can be employed to search for virulence factors in bacterial pathogens. Furthermore, MGK is not limited to assessing the fitness contributions of protein-encoding genes; the methodology can easily be adapted to study the effects of different chromosomal alterations, including inactivation of noncoding RNAs and gene-controlling elements. Overall, MGK can serve as a powerful experimental tool for any micro-organism in which global mutagenesis can be performed.

## Materials and methods

### Generation of microarray targets

MGK microarray target preparation includes six steps: preparation of genomic DNA, linear amplification of single-stranded DNA flanking the gene-inactivation cassette, separation of single-stranded flanks from genomic DNA, polyadenylation of the 3'-ends of single-stranded flanks, PCR amplification of the DNA flanks, and fluorescent dye conjugation. Individual steps are described below in detail. Parameters optimized for MGK microarray target preparation are shown in Additional data file 6.

#### Preparation of genomic DNA

After each selection, cells were harvested and genomic DNA was isolated from approximately 10^11 ^cells using cetyltrimethyl ammonium bromide protocol, as described by Ausubel [26].

#### Linear amplification of single-stranded DNA flanking the gene-inactivation cassette or the site of transposon insertion

For the defined library, the primer extension reactions were carried out in 100 μl of 1× HotMaster PCR reaction buffer (Eppendorf, Hamburg, Germany) containing 2.5 mmol/l MgCl_2_, 0.4 mmol/l of each deoxynucleoside triphosphate, 40 μg of genomic DNA (the equivalent of 8 × 10^9 ^*E. coli *genomes), 2 pmol of each of outward-facing biotinylated primers (Up-BIO and Dn-BIO in Table 1, corresponding to upstream and downstream ends of the gene disruption cassette, respectively), and 10 U HotMasterTaq DNA polymerase (Eppendorf). Generation of flanks from the random library was carried out under the same conditions, except 4 pmol of a single outward-facing primer Tn10OE-BIO (complementary to inverted repeats of Tn10 transposon) was used. The reactions were heated at 94°C for 2 minutes, followed by 15 cycles of 94°C for 30 s and 60°C for 20 s. All of the following experimental steps are performed at room temperature unless otherwise noted.

#### Purification of amplified single-stranded flanks

The amplified biotinylated flanks were separated from the genomic DNA using streptavidin-coated magnetic beads. Before use, 15 μl (150 μg) of M-270 streptavidin-coated magnetic Dynabeads^® ^(Invitrogen, Carlsbad, CA, USA) were washed twice with 200 μl binding and washing (BW) buffer (1 mol/l NaCl, 5 mmol/l Tris-HCl [pH 7.5], 0.5 mmol/l EDTA). The primer extension reaction was mixed with an equal volume of 2× BW buffer, and the mixture was added directly to the washed Dynabeads and incubated for 15 minutes to allow attachment of biotinylated DNA. Beads containing biotinylated DNA flanks were separated from the supernatant using the MPC^®^-S magnetic rack (Invitrogen), re-suspended in 200 μl BW buffer, and transferred to a new microcentrifuge tube. After removal of the buffer, beads were resuspended in 200 μl of 50% formamide, and separated from supernatant using the magnetic rack. The wash with 50% formamide was repeated five times. Beads were then washed three times with 200 μl H_2_O and finally resuspended in 20 μl H_2_O (equivalent to 100 μl of primer extension reaction).

#### Polyadenylation of the 3'-ends of single-stranded DNA

The reaction was carried out in a total volume of 50 μl of buffer no. 4 (New England Biolabs, Ipswich, MA, USA; 50 mmol/l potassium acetate, 20 mmol/l Tris-acetate, 10 mmol/l magnesium acetate, and 1 mmol/l dithiothreitol) containing 0.25 mmol/l CoCl_2_, 60 μmol/l dATP, 20 U terminal deoxynucleotidyl transferase (New England Biolabs), and 20 μl of bead suspension from the previous step. The reaction was incubated for 1 hour at 37°C with shaking at 1000 rpm in an Eppendorf Thermomixer^®^, followed by 10 minutes heat inactivation at 75°C. Beads carrying polyadenylated DNA were separated from the supernatant, washed with 200 μl H_2_O, and resuspended in 20 μl of H_2_O.

#### PCR amplification of the DNA flanks

The polyadenylated bead-bound DNA was used as template for nested PCR amplification, with incorporation of amino-allyl dUTP allowing conjugation to fluorophores. To minimize cross-hybridization of the products amplified from the control and experimental DNA, unique mismatches were introduced into each set of nested PCR primers (Table 1). A pair of primers in each set contained one mismatch positioned five nucleotides away from the mismatch in the other set. One set of primers was used for amplification of targets to be labeled with Alexa Fluor 555, and the other set with Alexa Fluor 647 (Invitrogen). The 100 μl nested PCR reaction contained 0.2 mmol/l of each of dATP, dCTP, and dGTP; 80 μmol/l of dTTP; 120 μmol/l of amino allyl dUTP; 16 mmol/l (NH_4_)_2_SO_4_; 67 mmol/l Tris-HCl (pH 8.8); 0.01% Tween-20; 1.5 mmol/l MgCl_2_; 5 U Taq DNA polymerase (CLP, San Diego, CA, USA); and 2 μl of bead suspension from the previous step. For the defined library, the PCR reaction mixture contained 1 μmol/l each of the primers Up-cy3, Dn-cy3, and TATV-3 (for generating the targets to be labeled with Alexa Fluor 555). Alternatively, the mixture contained 1 μmol/l each of the primers Up-cy5, Dn-cy5, and TATV-5 (to generate the targets to be labeled with Alexa Fluor 647). For the random library, targets to be labeled with Alexa Fluor 555 were amplified with primers TATV-3 and Tn10OE OUT-Cy3; targets to be labeled with Alexa Fluor 647 were amplified with primers TATV-5 and Tn10OE OUT-Cy5 (final concentration of 1 μmol/l for each primer). PCR conditions were as follows: 94°C for 5 minutes followed by 30 cycles of 95°C for 10 s, 47°C for 10 s, and 68°C for 10 s. Each 100 μl nested PCR reaction yielded 3 to 5 μg of target DNA product, with an average size of 300 bp. Usually, four to five 100 μl PCR reactions were performed. Amino-allyl modified PCR products were purified using the Wizard SV Gel and PCR Clean-up system following the manufacturer's protocol (Promega, Madison, WI, USA) and concentrated by ethanol precipitation.

#### Fluorescent dye conjugation

PCR product (10 to 20 μg) from the previous step was used for conjugation with Alexa Fluor 555 or Alexa Fluor 647 dyes, following the manufacturer's protocol (Invitrogen). Labeled products were purified with the Wizard SV Gel and PCR Clean-up system and concentrated by ethanol precipitation. DNA concentration and dye incorporation were determined using a NanoDrop^® ^ND-1000 UV/Vis spectrophotometer (NanoDrop Technologies, Wilmington, DE, USA).

### DNA microarrays

CombiMatrix™ DNA microarrays (CombiMatrix Corporation, Mukilteo, WA, USA) were custom designed for the detection of knockout mutants in the defined or the random library. For the defined gene-replacement library, microarray probes represented the chromosomal regions adjacent to the deletion cassette. To select these probe sequences, CombiMatrix™ DNA microarray design software scanned for 32-36-mer sequences within 100 bp regions upstream and downstream of each gene [27], and a total of 8,942 probes was synthesized on the microarray. For the random transposon insertion library, the microarray contained 11,579 unique 32-36-mer oligonucleotide probes, spaced approximately every 500 bp in the *E. coli *genome. Both types of microarrays also contained about 500 negative control probes corresponding to *Arabidopsis thaliana*, *Agrobacterium tumifaciens*, and phage lambda DNA sequences. Additional data files 1 and 2 contain information about the microarray design and oligonucleotide sequences of probes for each library.

### Microarray hybridization

CombiMatrix™ microarrays were hybridized with 5 μg each of differentially labeled target DNA for 20 hr at 50°C in a rotating oven. All hybridization and washing steps were performed in accordance with the manufacturer's protocol.

### Data acquisition and analysis

Microarrays were scanned using a PerkinElmer confocal dual-laser microarray scanner equipped with ScanArray Lite software (PerkinElmer, Boston, MA, USA). CombiMatrix Microarray Imager™ analysis software was used to obtain raw signal intensities. After background subtraction, intensity values were initially normalized on the basis of individual contribution to the total intensity of the channel. These values then underwent a second normalization based on contribution to intensity within a range of 50 probes upstream and downstream of each probe using custom designed Python software (available upon request). This second, 'sliding scale' normalization method accounted for the localized variation in DNA copy number caused by the varying rates of chromosome replication between the two conditions [28,29]. Resulting signal intensities for probes were used to calculate the intensity ratios (values in reference/values in selection). Intensity ratios were considered significant if they were greater than or equal to 3 in two independent experiments, with dye swapping.

### Bacterial strains, media, and growth conditions

The defined collection of *E. coli *gene replacement mutants [4] was obtained from Hirotada Mori (Nara Institute of Science and Technology, Nara, Japan). This collection is comprised of 3,985 *E. coli *deletion strains derived from wild-type BW25113 (F^- ^λ^- ^*rph-1 *Δ*araBAD*_AH33 _*lacI*^q ^Δ*lacZ*_WJ16 _*rrnB*_T14 _Δ*rhaBAD*_LD78 _*hsdR514*). In each deletion strain, the coding region (except for seven codons at the carboxyl-terminus) of a nonessential gene is replaced by in-frame insertion of a kanamycin resistance gene [30]. For application of MGK, individual deletion mutants were grown in 96-deep-well plates overnight at 37°C to an optical density (OD) of about 1.3 at 600 nm in Luria-Bertani (LB) medium containing 30 μg/ml kanamycin. An equal volume of each culture was combined. Cells were harvested, washed with LB, and re-suspended in LB supplemented with 15% glycerol. Aliquots containing about 1 × 10^9 ^cells in 500 μl were frozen and stored at -80°C.

### Construction of *E. coli *random transposon insertion library

The random transposon insertion library was generated using mini-Tn10 transposon mutagenesis. The suicidal transposon delivery vector pBSL177 was used, which contains a Tn10 transposon that harbors a kanamycin resistance marker [3], and a mutant transposase with altered target specificity controlled by an isopropyl-beta-D-thiogalactoside (IPTG) inducible *tac *promoter [31]. We first generated a random library of about 1 × 10^5 ^transposon mutants in *E. coli *cells grown in LB, but transposon insertions were severely biased toward chromosomal regions close to the origin of replication (data not shown). Therefore, in order to reduce this bias, transposition was induced in slowly grown cells (see Additional data file 1). Electrocompetent wild-type *E. coli *MG1655 cells were prepared from a culture grown in M9 minimal medium supplemented with 0.2% sodium acetate and electrotransformed with the pBSL177 plasmid (1 μg; 1.7 kV, 200 Ω resistance, and 25 μF capacitance). Cells were diluted with 1 ml SOB medium (2% tryptone, 0.5% yeast extract, 10 mmol/l NaCl, 2.5 mmol/l KCl, 10 mmol/l MgCl_2_, and 10 mmol/l MgSO_4_) containing 1 mmol/l IPTG, incubated with shaking at 37°C for 30 minutes, and plated on LB agar containing 30 μg/ml kanamycin. The next day, about 1.2 × 10^5 ^transformants were pooled, washed with LB, and re-suspended in 15% glycerol LB medium. Aliquots containing about 2 × 10^9 ^cells in 500 μl were frozen and stored at -80°C.

### Aromatic amino acid selection

All incubations were performed at 37°C with shaking. A glycerol stock of the mixed library (random or defined) was inoculated to OD_600 _0.02 into Neidhardt supplemented MOPS-defined medium [32] containing aromatic amino acids (0.4 mmol/l phenylalanine, 0.1 mmol/l tryptophan, and 0.2 mmol/l tyrosine) and grown overnight. Cultures were then diluted 100-fold in the same medium and grown to OD_600 _0.4. Cells were washed in 1× MOPS-Tricine buffer (pH 7.4; Teknova, Hollister, CA, USA), re-suspended in the same buffer, and inoculated into two flasks: one containing Neidhardt supplemented MOPS-defined medium complete with aromatic amino acids and the other containing the same medium but without aromatic amino acids. The starting density of the cultures was OD_600 _0.002. Cultures were grown to OD_600 _2, at which point the cells were collected and genomic DNA was isolated. The same conditions were applied for validation of individual mutant strains from the defined mutant library.

### Chloramphenicol selection

Minimum inhibitory concentration of chloramphenicol for wild-type BW25113 cells was determined to be 8 μg/ml. For chloramphenicol selection, a glycerol stock of the mixed defined library was inoculated to OD_600 _0.02 in 200 ml LB and grown to OD_600 _0.2, at which point the culture was split into two flasks. One of the flasks was supplemented with 80 μg/ml chloramphenicol, whereas the other flask served as a control. The chloramphenicol-containing culture was incubated for 18 hours with shaking at 37°C. The cells were harvested, washed with 100 ml LB, re-suspended in 100 ml LB at OD_600 _0.02, and grown to OD_600 _0.2. At this point chloramphenicol was again added at 80 μg/ml, and the culture was incubated for an additional 18 hours. After the second round of chloramphenicol selection, cells were harvested, washed with LB, re-suspended in LB to OD_600 _0.1, and grown to OD_600 _3. Cells were then harvested and genomic DNA was isolated. The control culture was grown to OD_600 _0.4 and diluted into fresh LB to OD_600 _0.02. After growing to OD_600 _0.4, the culture was diluted to OD_600 _0.1 and grown to OD_600 _3, at which point cells were harvested for genomic DNA isolation. For phenotype verification, individual mutants from the defined library were grown to OD_600 _0.2, at which point chloramphenicol was added at 80 μg/ml and incubated for 18 hours. The number of viable cells was determined at zero time point and after chloramphenicol treatment.

## Additional data files

The following additional data files are available with the online version of this paper. Additional data file 1 contains microarray raw data from the aromatic amino acid selection performed with the defined library and log ratios of probes calculated with normalized signal intensities. Additional data file 2 contains microarray raw data from the aromatic amino acid selection performed with the random library and log ratios of probes calculated with normalized signal intensities. Additional data file 3 contains microarray raw data from the chloramphenicol selection performed with the defined library and log ratios of probes calculated with normalized signal intensities. Additional data file 4 illustrates the biased representation of transposon mutants in the random transposon insertion library used in this study. Additional data file 5 shows the chromosomal location of genes identified in the aromatic amino acid selection with the defined and random libraries. Additional data file 6 shows optimized parameters of microarray target preparation in MGK.

## Supplementary Material

Additional data file 1 Presented is a table containing microarray raw data from the aromatic amino acid selection performed with the defined library and log ratios of probes calculated with normalized signal intensities. Click here for file

Additional data file 2 Presented is a table containing microarray raw data from the aromatic amino acid selection performed with the random library and log ratios of probes calculated with normalized signal intensities. Click here for file

Additional data file 3 Presented is a table containing microarray raw data from the chloramphenicol selection performed with the defined library and log ratios of probes calculated with normalized signal intensities. Click here for file

Additional data file 4 Presented is a figure illustrating the biased representation of transposon mutants in the random transposon insertion library used in this study. Click here for file

Additional data file 5 Presented is a figure showing the chromosomal location of genes identified in the aromatic amino acid selection with the defined and random library. Click here for file

Additional data file 6 Presented is a figure showing optimized parameters of microarray target preparation in MGK. Click here for file

## References

[B1] Riley M, Abe T, Arnaud MB, Berlyn MKB, Blattner FR, Chaudhuri RR, Glasner JD, Horiuchi T, Keseler IM, Kosuge T (2006). *Escherichia coli *K-12: a cooperativelydeveloped annotation snapshot: 2005.. Nucleic Acids Res.

[B2] Pittard AJ (1996). Biosynthesis of the aromatic amino acids.. Escherichia coli and Salmonella: Cellular and Molecular Biology.

[B3] Alexeyev MF, Shokolenko IN (1995). Mini-Tn10 transposonderivatives for insertion mutagenesis and gene delivery into the chromosome of Gram-negative bacteria.. Gene.

[B4] Baba T, Ara T, Hasegawa M, Takai Y, Okumura Y, Baba M, Datsenko KA, Tomita M, Wanner BL, Mori H (2006). Construction of *Escherichia coli *K-12 in-frame, single-gene knockout mutants: the Keio collection.. Mol Syst Biol.

[B5] Zhao G, Pease A, Bharani N, Winkler M (1995). Biochemical characterization of *gapB*-encoded erythrose 4-phosphate dehydrogenase of *Escherichia coli *K-12 and its possible role in pyridoxal 5'-phosphate biosynthesis.. J Bacteriol.

[B6] Zhao G, Winkler M (1994). An *Escherichia coli *K-12 *tktA tktB *mutant deficient in transketolase activity requires pyridoxine (vitamin B_6_) as well as the aromatic amino acids and vitamins for growth.. J Bacteriol.

[B7] Sprenger G (1995). Genetics of pentose-phosphate pathway enzymes of *Escherichia coli *K-12.. Arch Microbiol.

[B8] Vázquez D (1979). Inhibitors of Protein Biosynthesis.

[B9] Sassetti CM, Boyd DH, Rubin EJ (2001). Comprehensive identification of conditionally essential genes in mycobacteria.. Proc Natl Acad Sci USA.

[B10] Badarinarayana V, Estep III PW, Shendure J, Edwards J, Tavazoie S, Lam F, Church GM (2001). Selection analyses of insertional mutants using subgenic-resolution arrays.. Nat Biotechnol.

[B11] Lamichhane G, Tyagi S, Bishai WR (2005). Designer arrays for defined mutant analysis to detect genes essential for survival of *Mycobacterium tuberculosis *in mouse lungs.. Infect Immun.

[B12] Hensel M, Shea JE, Gleeson C, Jones MD, Dalton E, Holden DW (1995). Simultaneous identification of bacterial virulence genes bynegative selection.. Science.

[B13] Tam C, Glass EM, Anderson DM, Missiakas D (2006). Transposon mutagenesis of *Bacillus anthracis *strain Sterne using *Bursa aurealis*.. Plasmid.

[B14] Kobayashi K, Ehrlich SD, Albertini A, Amati G, Andersen KK, Arnaud M, Asai K, Ashikaga S, Aymerich S, Bessieres P (2003). Essential *Bacillus subtilis *genes.. Proc Natl Acad Sci USA.

[B15] Shin SJ, Wu C-W, Steinberg H, Talaat AM (2006). Identificationof novel virulence determinants in *Mycobacterium paratuberculosis *by screening a library of insertional mutants.. Infect Immun.

[B16] Geoffroy M-C, Floquet S, Metais A, Nassif X, Pelicic V (2003). Large-scale analysis of the meningococcus genome by genedisruption: resistance to complement-mediated lysis.. Genome Res.

[B17] Jacobs MA, Alwood A, Thaipisuttikul I, Spencer D, Haugen E, Ernst S, Will O, Kaul R, Raymond C, Levy R (2003). Comprehensive transposon mutant library of *Pseudomonas aeruginosa*.. Proc Natl Acad Sci USA.

[B18] Liberati NT, Urbach JM, Miyata S, Lee DG, Drenkard E, Wu G, Villanueva J, Wei T, Ausubel FM (2006). An ordered, nonredundant library of *Pseudomonas aeruginosa *strain PA14 transposon insertionmutants.. Proc Natl Acad Sci USA.

[B19] Bae T, Banger AK, Wallace A, Glass EM, Aslund F, Schneewind O, Missiakas DM (2004). *Staphylococcus aureus *virulence genesidentified by *bursa aurealis *mutagenesis and nematodekilling.. Proc Natl Acad Sci USA.

[B20] Winzeler EA, Shoemaker DD, Astromoff A, Liang H, Anderson K, Andre B, Bangham R, Benito R, Boeke JD, Bussey H (1999). Functional characterization of the *Saccharomyces cerevisiae *genome by gene deletion and parallel analysis.. Science.

[B21] Ramer J, Bell R (1990). Expression of the phospholipid-dependent *Escherichia coli *sn-1,2-diacylglycerol kinase in COS cells perturbs cellular lipid composition.. J Biol Chem.

[B22] Sugawara E, Nikaido H (1992). Pore-forming activity of OmpAprotein of *Escherichia coli*.. J Biol Chem.

[B23] Hara H, Yamamoto Y, Higashitani A, Suzuki H, Nishimura Y (1991). Cloning, mapping, and characterization of the *Escherichia coli prc *gene, which is involved in C-terminal processing of penicillin-binding protein 3.. J Bacteriol.

[B24] Iuchi S, Lin EC (1992). Purification and phosphorylation of the Arc regulatory components of *Escherichia coli*.. J Bacteriol.

[B25] Storz G, Jacobson FS, Tartaglia LA, Morgan RW, Silveira LA, Ames BN (1989). An alkyl hydroperoxide reductase induced by oxidative stress in *Salmonella typhimurium *and *Escherichia coli*: genetic characterization and cloning of *ahp*.. J Bacteriol.

[B26] Ausubel FM (1994). Current Protocols in Molecular Biology.

[B27] CombiMatrix. http://www.combimatrix.com/support_faqhtm#whatsteps.

[B28] Cooper S, Helmstetter CE (1968). Chromosome replication and the division cycle of *Escherichia coli *B/r.. J Mol Biol.

[B29] Sherratt DJ (2003). Bacterial chromosome dynamics.. Science.

[B30] Datsenko KA, Wanner BL (2000). One-step inactivation of chromosomal genes in *Escherichia coli *K-12 using PCR products.. Proc Natl Acad Sci USA.

[B31] Bender J, Kleckner N (1992). IS10 transposase mutations that specifically alter target site recognition.. EMBO J.

[B32] Neidhardt FC, Bloch PL, Smith DF (1974). Culture medium for enterobacteria.. J Bacteriol.

