# Supplementary material for: Monitoring of gene knockouts: genome-wide profiling of conditionally essential genes
Source: Genome Biol. 2007 May 22;8(5):R87. doi: 10.1186/gb-2007-8-5-r87 (PMC1929150; doi:10.1186/gb-2007-8-5-r87)
Supplement: Additional data file 4 — Presented is a figure illustrating the biased representation of transposon mutants in the random transposon insertion library used in this study. [file gb-2007-8-5-r87-S4.pdf]

Supplementary Figure 1

Biased representation of transposon mutants in a random transposon insertion library. Custom-designed CombiMatrix DNA microarrays were hybridized with MGK targets generated from **(a)** the random transposon library of 1.2x 10^5 mutants, and **(b)** the defined library (3,985 mutants in the Keio collection) of equally mixed mutants. Microarray designs for each type of library are described in Materials and methods. In both types of microarrays, the probes are ordered sequentially, beginning from the top left at 0 min on the *E. coli* chromosome and proceeding from left to right, top to bottom. The entire chromosome is represented by the probes in the top 2/3 of the chip, and the bottom 1/3 of each chip contains repeats of every 4th probe. **(a)** In the microarray hybridized with the MGK targets prepared from the random library, probes corresponding to regions near the replication terminus (indicated by brackets) show weak or no hybridization. **(b)** The MGK targets prepared from the defined library exhibit a more uniform hybridization. This result indicates that despite a high complexity in the random library ($1.2x10^5$ transposon mutants), transposon insertions in genes proximal to the replication terminus (*ter*) are represented less than those in genes close to the replication origin (*ori*). It should be noted that the random library used in (a) was generated in slowly-grown cells (see Materials and methods). A previous random library ($1x10^5$ mutants) generated using rapidly-grown cells in LB medium displayed an even more pronounced bias than one shown in (a) (data not shown).

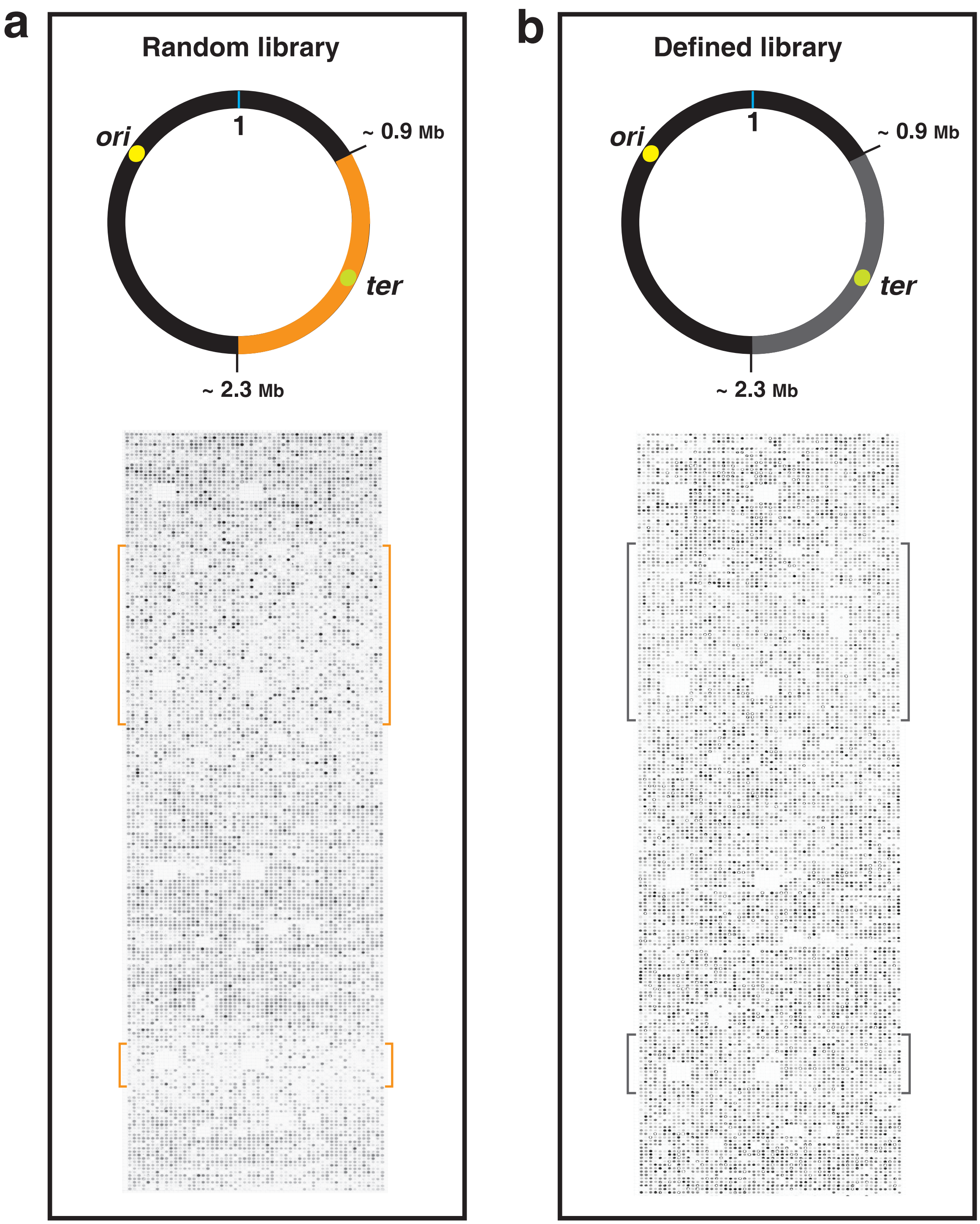
a
Random library
ori
1
~ 0.9 Mb
ter
~ 2.3 Mb
b
Defined library
ori
1
~ 0.9 Mb
ter
~ 2.3 Mb
