# Supplementary material for: Monitoring of gene knockouts: genome-wide profiling of conditionally essential genes
Source: Genome Biol. 2007 May 22;8(5):R87. doi: 10.1186/gb-2007-8-5-r87 (PMC1929150; doi:10.1186/gb-2007-8-5-r87)
Supplement: Additional data file 5 — Presented is a figure showing the chromosomal location of genes identified in the aromatic amino acid selection with the defined and random library. [file gb-2007-8-5-r87-S5.pdf]

Supplementary Figure 2

Chromosomal location of genes identified in the aromatic amino acid selection with the defined (red) and random (black) library. The start position of each gene on the chromosome is as follows: *aroA* (959,234); *trpA* (1,318,936); *trpB* (1,320,129); *trpC* (1,321,499); *trpD* (1,323,098); *trpE* (1,324,660); *aroD* (1,776,400); *aroC* (2,452,919); *pheA* (2,736,401); *tyrA* (2,738,725); *aroB* (4,121,930); *aroE* (4,209,578); and *tyrB* (4,270,704). That most genes unidentified with the random library are located around the replication terminus region indicates that mutants corresponding to those genes are underrepresented in the random library.

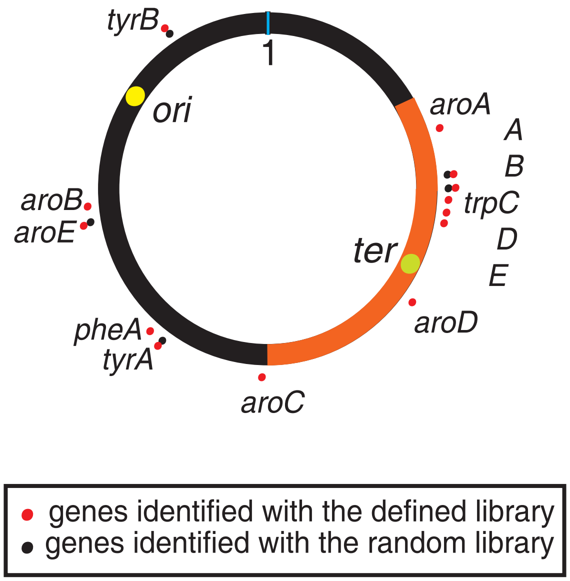

tyrB
1
ori
aroA
A
B
trpC
D
E
aroB
aroE
ter
aroD
pheA
tyrA
aroC
genes identified with the defined library
genes identified with the random library
