# Supplementary material for: Monitoring of gene knockouts: genome-wide profiling of conditionally essential genes
Source: Genome Biol. 2007 May 22;8(5):R87. doi: 10.1186/gb-2007-8-5-r87 (PMC1929150; doi:10.1186/gb-2007-8-5-r87)
Supplement: Additional data file 6 — Presented is a figure showing optimized parameters of microarray target preparation in MGK. [file gb-2007-8-5-r87-S6.pdf]

Supplementary Figure 3

Optimization of microarray target preparation in MGK. (**a**) Determination of the optimum number of cycles for linear amplification of single-stranded DNA. A 50 μl of reaction mixture contains 40 μg of genomic DNA (~12 fmole) isolated from the random library; 2 μl, 10 mM dNTP; 3 pmole, ^{32}P-labeled Tn10OE primer; and 2 μl, Taq DNA polymerase (HotMaster, Eppendorf) in 1x HotMaster PCR buffer. Reactions were carried out at 94°C for 30 sec and 60°C for 20 sec, and samples (6 μl) were taken each at 15, 30, 45, and 60th cycle to run on 6% SDS-polyacrylamide gel with 8 M urea. We chose the minimum number of cycles, 15 cycles, to prevent a possible appearance of any dominant species of single-stranded DNA. (**b**) Determination of the optimum molar ratio of primer to genome for production of single-stranded DNA. ^{32}P-labeled Tn10OE primer was added at the ratio (primer to genome) of 100, 300, 500, 700 or 1000 (lanes 1-5), respectively. Reaction was carried out for 15 cycles of 94°C for 30 sec and 60°C for 20 sec. A 25 μl of reaction mixture contains the same as in (a) except 10 μg of genomic DNA (~3 fmole) used as template and varying primer concentration. Samples (4 μl) were run on 6% SDS-polyacrylamide gel with 8 M urea. We chose the ratio, 300, of primer to genome to prevent a possible appearance of any dominant species of single-stranded DNA. (**c**) A summary of different washing systems tested. The biotinylated flanks were produced in 100 μl of primer extension reaction at the condition chosen in (a) and (b), and separated from the genomic DNA using streptavidin-coated magnetic beads. Beads containing biotinylated flanks were washed using each washing system and finally resuspended in 20 μl H_2O (equivalent to 100 ml of primer extension reaction). The beads (2 μl each) were then used as template in 50 μl of PCR for 30 cycles of 94°C for 30 sec,

55°C for 30 sec, and 68°C for 30 sec. As a control for PCR, wild-type genomic DNA was used as template to amplify an essential (*plsC*) and a nonessential gene (*yghG*), respectively. PCR amplification of the essential gene indicates the contamination of genomic DNA in the beads. (1) The beads were washed 5 times with 200 μl of 50% formamide followed by washing 5 times with water. (2) The beads were incubated at 55°C for 5 min in water before washing with water in (1). (3) The beads were incubated at 90°C for 10 min in 10 mM NaOH before washing with water in (1). (4) The beads were incubated at 90°C for 5 min in 1x HotMaster PCR buffer (Eppendorf) before washing with water in (1). M: DNA ladder. Samples (10 μl) were run on 1% agarose gel. We decided to use the washing system 1, in which loss of single-stranded DNA is minimal. (**d**) Size distribution of single-stranded DNA produced. A mutant from the random library was sequenced for transposon insertion site (*yhiL*) and used for the preparation of biotinylated single-stranded DNA with a biotinylated Tn10OE primer. With the biotinylated single-stranded DNA as template, PCR was performed with a kanamycin specific outward primer and primers designed for amplification of 300-800 bp DNA fragments of the *yhiL* gene, respectively. As a control, wild-type *E. coli* genomic DNA was used as template. Samples (10 μl) were run on 1% agarose gel. M: DNA ladder.

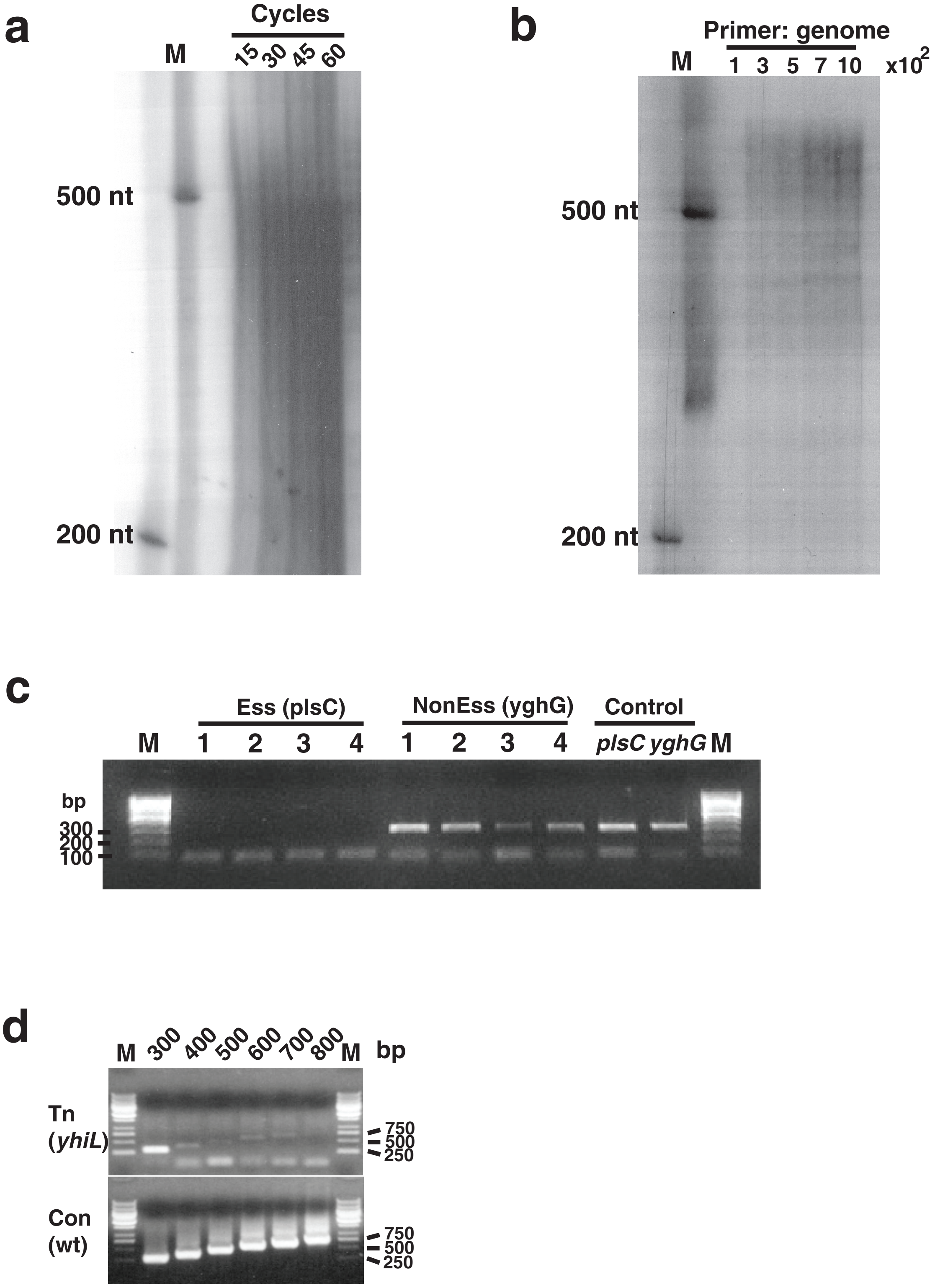

a
Cycles
M
15 30 45 60
500 nt
200 nt
b
Primer: genome
M
1 3 5 7 10
x10^2
500 nt
200 nt
c
Ess (plsC)
NonEss (yghG)
Control
M 1 2 3 4 1 2 3 4 *plsC* *yghG* M
bp
300
200
100
d
M 300 400 500 600 700 800 M bp
Tn (*yhiL*)
750
500
250
Con (wt)
750
500
250
